# Atomistic mechanisms of human TRPA1 activation by electrophile irritants through molecular dynamics simulation and mutual information analysis

**DOI:** 10.1038/s41598-022-08824-7

**Published:** 2022-03-23

**Authors:** Matthew Habgood, David Seiferth, Afroditi-Maria Zaki, Irfan Alibay, Philip C. Biggin

**Affiliations:** 1 Department of Biochemistry, University of Oxford, South Parks Road, Oxford, OX1 3QU UK; 2 AWE Aldermaston, Reading, Berkshire, RG7 4PR UK

**Keywords:** Molecular modelling, Computational biophysics

## Abstract

The ion channel TRPA1 is a promiscuous chemosensor, with reported response to a wide spectrum of noxious electrophilic irritants, as well as cold, heat, and mechanosensation. It is also implicated in the inception of itch and pain and has hence been investigated as a drug target for novel analgesics. The mechanism of electrophilic activation for TRPA1 is therefore of broad interest. TRPA1 structures with the pore in both open and closed states have recently been published as well as covalent binding modes for electrophile agonists. However, the detailed mechanism of coupling between electrophile binding sites and the pore remains speculative. In addition, while two different cysteine residues (C621 and C665) have been identified as critical for electrophile bonding and activation, the bound geometry has only been resolved at C621. Here, we use molecular dynamics simulations of TRPA1 in both pore-open and pore-closed states to explore the allosteric link between the electrophile binding sites and pore stability. Our simulations reveal that an open pore is structurally stable in the presence of open ‘pockets’ in the C621/C665 region, but rapidly collapses and closes when these pockets are shut. Binding of electrophiles at either C621 or C665 provides stabilisation of the pore-open state, but molecules bound at C665 are shown to be able to rotate in and out of the pocket, allowing for immediate stabilisation of transient open states. Finally, mutual information analysis of trajectories reveals an informational path linking the electrophile binding site pocket to the pore via the voltage-sensing-like domain, giving a detailed insight into the how the pore is stabilized in the open state.

## Introduction

TRPA1 (transient receptor potential ankyrin 1, or transient receptor potential channel subfamily A member 1) is a homotetrameric, Ca^2+^-permeable membrane protein that is the subject of diverse current research interest^1,2^. It is well-established as a chemosensor activated by noxious electrophile irritants, of which the best known examples are perhaps allyl isothiocyanate (AITC), the active ingredient of wasabi and mustard oil, and cinnamaldehyde, giving rise to a burning sensation^3,4^; it is separately activated by a set of non-electrophilic compounds^5,6^, of which the best-known is menthol^7^, giving rise to a cold sensation. In species other than *homo sapiens* the channel is also known to act as a mechanosensor, as well as a sensor of cold and heat, but the ability of human TRPA1 to fulfil these roles remains controversial^8–12^. The channel is additionally activated by, and may act as a sensor for, acidity (protons)^13^ and calcium^14–16^. TRPA1 is expressed in nociceptive neurons^17^, where it acts directly in the inception of pain and itch, but has also been found in the vascular endothelium^18^ and lung epithelium^19^ among others places. Significantly, it has been shown to be activated by endogenous inflammation messengers such as bradykinin^19^ and nitrooleic acid^20^ (as well as miRNAs^21^), and hence appears to play a role in inflammation of critical tissues^22,23^.

Given the polymodal sensing behaviour and diverse involvement in nociception and signalling, it is unsurprising that the last decade has seen widespread efforts to develop modulators for TRPA1, with an ultimate goal of producing approved pharmaceuticals. In addition to purpose-designed high-activity electrophile agonists^24^, the range of non-electrophilic agonists has been expanded^24^, and even lipid raft disruptors have been shown to have activity^25^. Photosensitive ligands have also been used to add light-sensitivity to TRPA1^26,27^. However, the greatest activity has been in the development of antagonists^28–30^. A primary clinical goal for TRPA1 modulation has been novel analgesics^31^, but developmental drugs based on TRPA1 antagonism that have reached phase II clinical trials have also been aimed at neuropathy and respiratory disorders^32^. Recent efforts have also aimed at the development of TRPA1 modulators for the treatment of cardiovascular disease^33^ and chronic itch^34^. Applications in skin regeneration have even been explored^35^.

As complete as possible a mapping of the mechanisms of activation of TRPA1 is therefore of importance both for fundamental understanding of the body’s signalling apparatus, and directly in ongoing efforts to develop new medicines. An obvious starting point is to determine the mechanism of activation by electrophilic irritants, which is the basis of the channel’s chemosensing behaviour. Initial experimental studies using mutagenesis^36^ indicated that these compounds formed covalent bonds with cysteine residues, and suggested that three key cysteine residues—C621, C641, and C665—were essential for activation by electrophile irritants. Some evidence was also presented that lysine residue K710 could mediate some electrophile-induced activation in the absence (mutation to a non-nucleophilic residue) of all three critical cysteines. More recent studies using the tag compound iodoacetamide (IAC) have clarified and modified these conclusions^37^. Mutagenesis in this later work indicated that C641 is not related to activation. The importance of C621 was confirmed, with electrophile activation completely extinguished by the C621A mutation; it was additionally shown that reaction of electrophiles with C621 is very rapid, occurring at ‘a much faster rate’ than with glutathione, the body’s natural scavenger of electrophiles, and > 6000 times faster than with nonreactive cysteine residues. The role of C665 was found to be more complicated. Under conditions for which C621 residues were found to be completely reacted with IAC, C665 residues were only 3–4% reacted. Furthermore, systems in which C665 reactivity was extinguished via C665L mutation were found to be non-responsive to electrophiles, despite full reaction of C621. The implication was noted that, at least for small electrophiles such as iodoacetamide, bonding at both C665 and C621 is necessary for channel activation. However, apparently paradoxically, it appears that C665 bonding is only required at some channels in order for all channels to be activated, since complete activation is achieved despite only 3–4% occupancy at C665. Very recent mutagenesis work has slightly modified these conclusions, suggesting that 30% electrophile activation is retained under the mutation C665S^38^.

**Figure 1:**
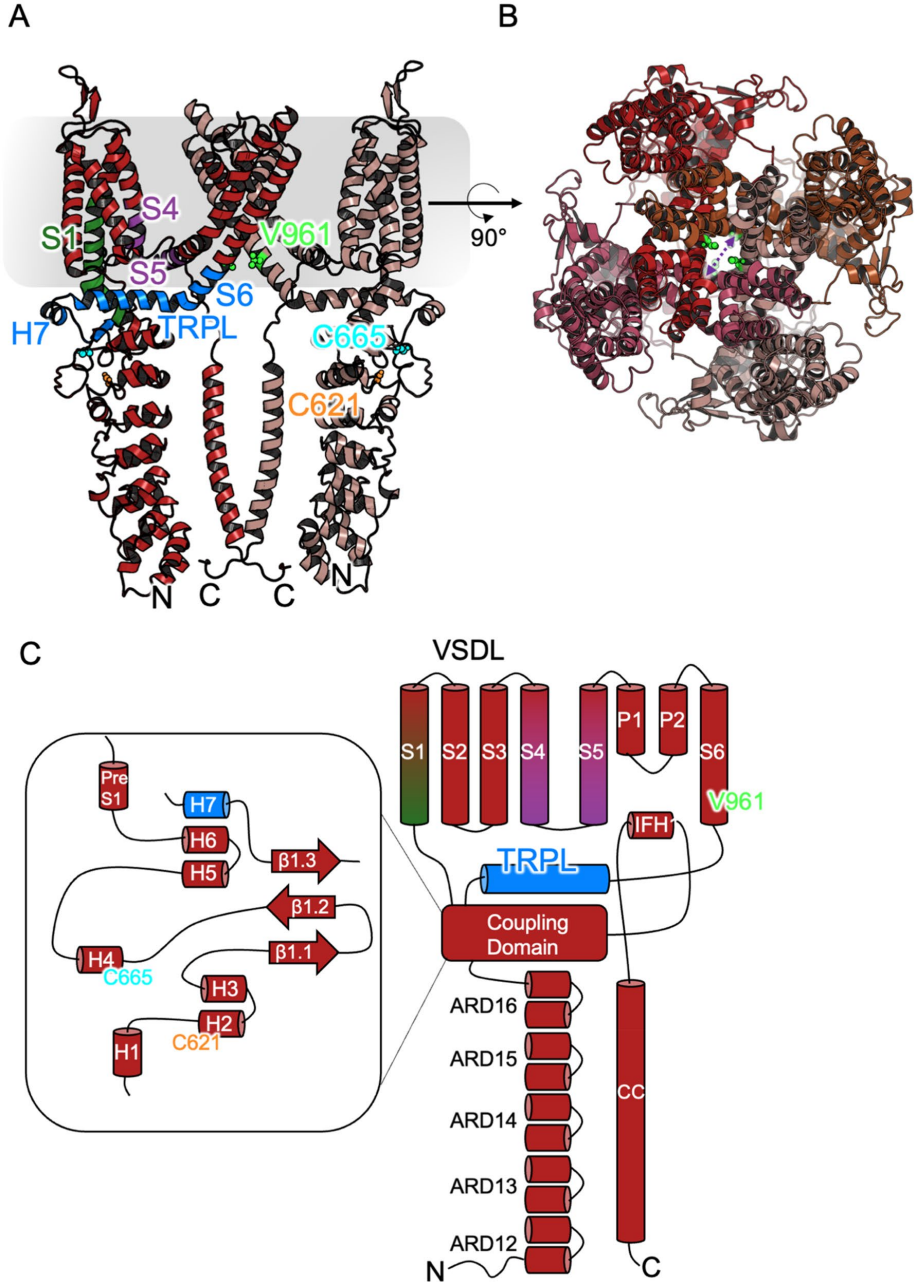
The structure of TRPA1 as determined by cryo EM. (**A**) Cartoon representation of the pore-closed structure 6PQQ, prepared for simulation (see “Methods”, Structure Preparation). Two monomer units are visible, coloured red and “chocolate” and the approximate position of the membrane is indicated by the grey rectangle. Residues V961 (green), C621 (orange) and C665 (cyan) are indicated as vdW spheres. Segments on chain A that are used in the mutual information analysis are coloured blue (V961-V1005, loop above pocket lid (H7), TRP-like (TRPL) domain and transmembrane domain (TMD) helix (part of S6)), green (Y706-L730, voltage-sensor like domain (VSLD) helix threaded through loop above pocket lid (S1)), and purple (Y842-V865 (VSLD helix (S4)) and connected TMD helix immediately adjoining V961 (S5)). ARD is short for ankyrin repeat domain, IFH is the interfacial helix, and CC indicates coiled-coil. (**B**) TRPA1 rotated through 90° looking down the channel pore and showing the constriction at the V961 residues, (**C**) Topology cartoon of TRPA1 (adapted from Suo et al.^40^).

Further understanding has come from two sets of cryogenic electron-microscopy (cryo EM) structures released in two very recent studies (PDB^39^ codes 6PQO-Q and 6VWX-Z) ^38,40^. These structures have revealed a general shape of TRPA1 and the arrangement of its domains (Fig. 1).

For electrophilic activation, C621 and C665 were revealed to be on the lower and upper lid (respectively) of a pocket found in the cytosolic so-called ‘coupling domain’ (Fig. 1), with one pocket per monomer for a total of four ‘binding pockets’ in the assembled channel. In the earlier of the two structure sets^40^, the central channel pore is closed in all cases. In a structure with no ligand bound, all binding pockets were closed (6PQQ), whereas in two structures with ‘large’ ligands (similar to the dimensions of the pocket itself), binding was observed at C621 and all pockets were open (6PQO, 6PQP). In the more recent of the two structure sets^38^, along with further ligand-free pocket-closed pore-closed and ligand-bound pocket-open pore-closed structures (6V9V-W, 6V9Y), a pore-open structure was also reported (6V9X). This structure also had open pockets with the ligand IAC bound at C621. In this structure, electron density consistent with the size of IAC was observed in the region of C665, but with insufficient electron density to allow an IAC ligand be fully resolved; this is consistent with the low frequency of IAC bonding at C665 observed in earlier studies^37^. It has been proposed^38^ that in the case of IAC, which is significantly smaller than the dimensions of the pocket, binding at both cysteines is necessary to fill enough space to force the pocket into an open state. It may be noted, though, that this is not compatible with the observation that only a small occupancy of binding at C665 is necessary for full activation of the channel.

Taken together, the structures provide an outline mechanism for electrophile irritant activation of TRPA1: electrophiles react rapidly with C621 (and to a lesser extent with C665); this results in pocket opening, probably due to space filling effects; and pocket opening encourages, or is a prerequisite for, pore opening. However, these structures provide only a static snapshot of the channel and leave important questions still to be answered. The two most important questions are:

(i) how does an open pocket influence whether the pore is open or closed and
(ii) what is the role of bonding to C665? If filling space to keep the pocket open is all that is required of a bound electrophile, why are reactions with both C621 and C665 required for channel activation?

We address these question in this work via molecular dynamics (MD) simulations in conjunction with pairwise residue-by-residue mutual information pathway analysis. MD simulations have been demonstrated to provide useful insights on ion channel functionality, while mutual information pathways can indicate the connection between allosteric and active sites. In order to answer question (i), we used µs-length simulations of pocket-closed pore-closed (6PQQ), pocket-open pore-closed (6PQP), and a series of structures generated by ligand modification of the pocket-open pore-open structure 6V9X. The trajectories from the pocket-open pore-open and pocket-closed pore-closed structures were then analysed to identify mutual information pathways between the pore and the pocket.

**Figure 2:**
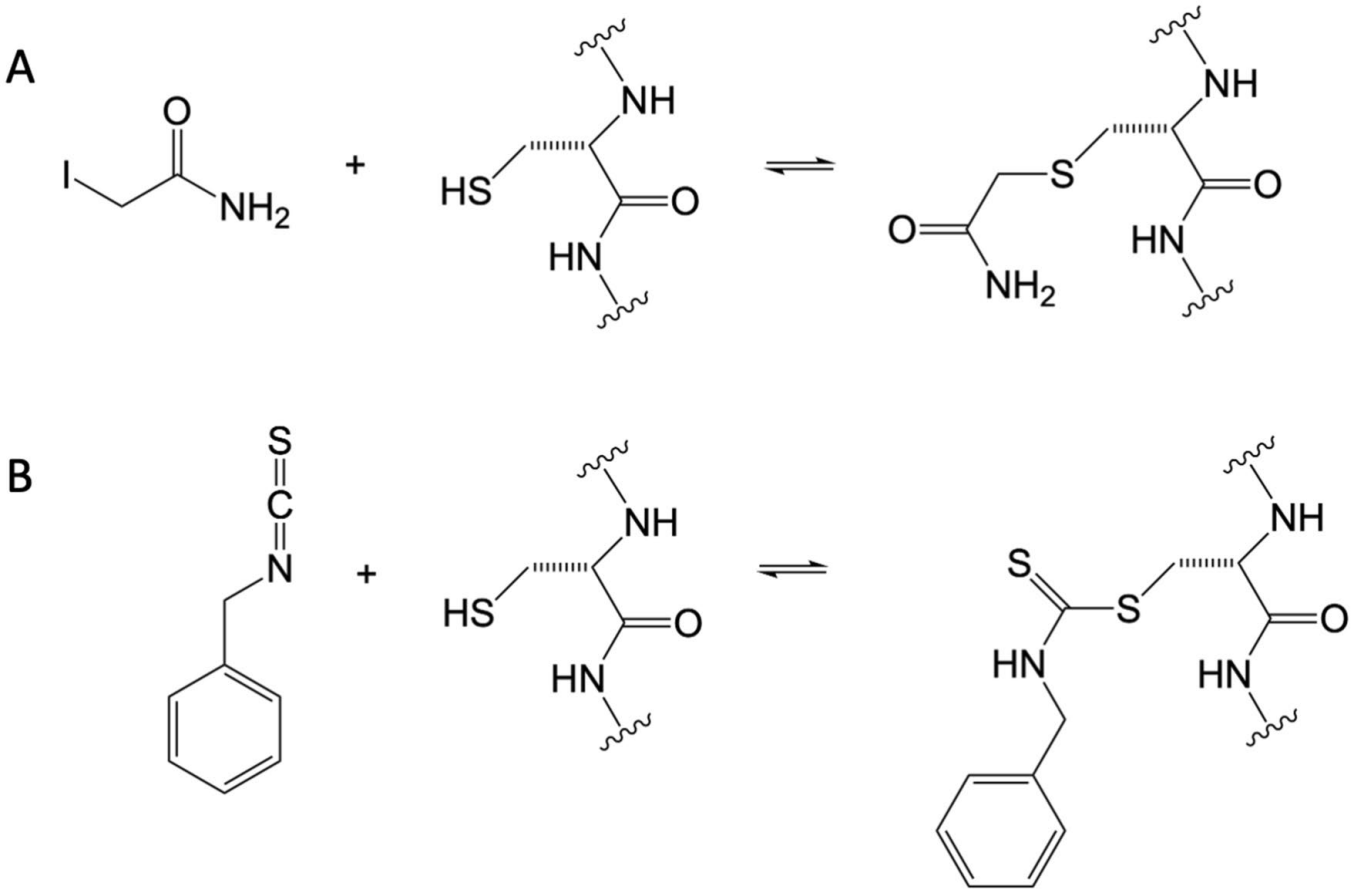
Molecular structures and cysteine bonding mechanisms of (**A**) iodoacetamide (IAC) and (**B**) benzyl isothiocyanate (BITC).

In order to answer question (ii), the series of simulated ligand modifications from 6V9X were re-analysed. These modifications comprised ligand deletion (no ligand); IAC bonded to C621; IAC bonded to C665; and, for comparison with a larger ligand of the same dimensions as the pocket, the benzyl isothiocyanate (BITC) bonded to C621, which was the label compound used experimentally in structure 6PQP. These molecules and their bonding mechanisms to cysteine are illustrated in Fig. 2. The simulations were analysed for changes in pocket width (openness), and pore width.

Previous work^41^ has presented an open state structure based on homology modelling, but focused on the role of the extracellular domain in stabilising the open-pore structure as opposed to the action of electrophiles at the binding pocket. Here we show that an open pore requires open pockets for structural stability, derived from a network of small structural shifts in the voltage-sensor-like-domain. We also demonstrate that covalently bound ligands (electrophiles) at C665 or at C621 drive the pocket towards open states and help to stabilise open states of the pore. In particular, IAC at C665 is shown to be able to rotate in and out of the pocket, although it is more stable within. Considering that C665 is solvent accessible when the pocket is fully closed, whereas C621 is not, this suggests that binding at C665 is important to initialize the pocket opening, particularly at short timescales.

## Methods

To explore the relationship between the geometry of the cytosolic coupling domain pocket in which C621 and C665 are found, with the channel pore, simulations were carried out on pocket-closed pore-closed, pocket-open pore-closed and pocket-open pore-open structures. The pocket-closed pore-closed and pocket-open pore-closed structures were taken directly from the cryo EM structures 6PQQ and 6PQP respectively. 6PQQ is a ligand-free (*apo*) structure, while 6PQP has benzyl isothiocyanate (BITC) bound at C621 within the open pocket. The pocket-open pore-open structures were generated from cryo EM structure 6V9X. To investigate the influence of the ligand, the binding scenario in 6V9X (IAC bound at C621) was modified in subsequent simulations to include ligand deletion (no ligand); IAC bound at C665; and BITC bound at C621. The full list of structures that were simulated is summarized in Table 1. In text that follows descriptions of the system are used, but if a PDB code is appended in brackets it refers to a simulation of one the unmodified or native structures (i.e. 6PQQ, 6PQP or 6V9X).

**Table 1:** Simulated structures and number of repetitions carried out for each simulation.

Condition	Simulation description
1	*Apo* pocket-closed pore-closed (6PQQ)
2	Pocket-open pore-closed with BITC ligand at C621 (6PQP)
3	Pocket-open pore-open with IAC ligand at C621 (6V9X)
4	*Apo* pocket-open pore-open (via ligand deletion from 6V9X)
5	Pocket-open pore-open with IAC ligand at C665 (via residue modification from 6V9X)
6	Pocket-open pore-open with BITC ligand at C621 (via residue modification from 6V9X)

All conditions were run 3 times (n) for 1 µs each. Where the simulation condition is simply the “native” state of the PDB structure, the PDB code is included in brackets after the description of the simulation in the text.

### Structure preparation

Cryo EM structures 6PQQ, 6PQP, and 6V9X were retrieved from the PDB. Preparation for simulation was carried out using the ‘Molecular Operating Environment’ (MOE)^42^. All of these structures had residues missing from the 3D structure, and significant numbers of residues for which backbone and C_β_ atoms were present but other side chain atoms were truncated (*i.e.* the residues appear as alanine in 3D). Backbone gaps were filled in using MOE’s ‘loop modeller’ module. This fills in missing structure segments, based on the residue sequence, by generating trial backbone geometries through random sampling from a distribution derived from the PDB. Generation is constrained by the position of the ‘anchor’ residues, the residues for which 3D coordinates are available at the edge of the gap^43^. Generated backbone geometries are filtered initially based on simple geometric criteria, and subsequently scored according to a proprietary function combining a coarse-grained energy model and a statistical scoring function that measures the loop candidate’s deviation from PDB average geometries. The top twenty scored backbone geometries were manually inspected. A single candidate was selected for further development. Side chain atoms were added in by sampling from MOE’s proprietary rotamer library, followed by structural optimisation and scoring under an AMBER10 force-field^44^ supplemented with extended Hückel theory (EHT)^45^ with the Generalized Born with Volume Integrals (GBVI) solvation model^46^. Missing sidechain atoms were added to residues truncated at C_β_ using the same procedure. Finally, missing hydrogen atoms were added using the ‘Protonate 3D’ module, assuming a pH of 7.4. This module adds hydrogen atoms at geometrically standardised positions. Residues with variable protonation states (Arg, Asp, Glu, Lys, His) and multiple tautomers (His, Asp, Glu) are resolved to particular states by sampling the different possibilities and evaluating them by an energy function based on AMBER10 supplement EHT with GBVI, supplemented by a penalty term for mismatches between the user-defined pH of the environment and the pK_a_ of the residue in a given state.

Ligands were docked, bonded to the appropriate cysteine as shown in Fig. 2 using MOE. London scoring (part of the MOE package) was used^42^. The top ten scored structures were examined, and a single pose selected by eye.

### Molecular dynamics simulation

The prepared TRPA1 structures were inserted into a model membrane consisting of a pre-equilibrated bilayer of 1-palmitoyl-2-oleoyl-*sn*-glycero-3-phosphocholine (POPC) lipid molecules. Insertion was carried out using scripts adapted from those available at the tutorial pages for GROMACS^47^. This resulted in 494 lipid molecules. Water and salt molecules were then added using GROMACS to give a rectangular simulation cell with at least 10 Å between any protein atom and the cell boundary, and with a salt concentration of 150 mM NaCl. The box was then neutralized via the addition of 8 Cl^−^ ions. This gave a unit cell of ~ 350,000 atoms, 244,299 of which were water. The system was parametrized using TIP3P for water^48^, AMBER99SB-ILDN^49^ for protein atoms, GAFF2 for ligand atoms with AM1-BCC charges^50^ generated in antechamber^51^, and Jambeck and Lyubartsev’s parameters for POPC^52,53^. Long-range electrostatics were handled using the smooth particle-mesh Ewald^54^ approach with a cut-off of 10 Å, and Lennard–Jones potentials were similarly cut off at 10 Å. Simulation cells were initially relaxed by 50,000 steps of steepest descent energy minimisation. Equilibration at a temperature of 310 K and a pressure of 1 atm was achieved with 5 ns simulation in an NVT ensemble and 10 ns in an NPT ensemble with all heavy protein atoms restrained with harmonic restraints at 1000 kJ mol^−1^ nm^−2^. Temperature was maintained using a Nose–Hoover thermostat^55^ and pressure was maintained using a Parrinello-Rahman barostat^56^. The LINCS algorithm was employed to constrain covalent bonds to hydrogen, allowing the use of a 2 fs time step^57^. Production run simulations were carried out for 1 µs and each condition run with three repeats as summarized in Table 1. Pore radii were computed using CHAP^58^.

### Calculation of pairwise mutual information

In order to determine the mechanism by which the opening of the binding pocket influences the opening and closing of the channel pore, pairwise mutual information was calculated between the residues in the section of the protein between the mobile pocket ‘lid’ and the pore itself, by analysis of the MD trajectories. Mutual information measures the degree to which motion of one residue is correlated with motion of another residue. The implementation used here was that used in LeVine et al.^59^ Specifically, the covariance between a spatial coordinate (*j* = *x, y,* or *z*) of atom *i* and spatial coordinate *l* of atom *k*, *X*_*i,j*_ and *X*_*k,l*_ respectively, is given by

1 $$C\left( {X_{i,j} * \, X_{k,l} } \right) = \left\langle {X_{i,j} *X_{k,l} } \right\rangle - \left\langle {X_{i,j} } \right\rangle *\left\langle {X_{k,l} } \right\rangle$$

*i.e.* the average across the molecular dynamics trajectory of the product of the two coordinates, minus the product of the averages of the two coordinates individually. Any combination (set) of coordinates, **X**, can then be used to calculate a configurational entropy *H*(**X**) over the corresponding set of atoms using the matrix of covariances, C(**X**):

2 $$H\left( {\mathbf{X}} \right) = \frac{1}{2}\ln \left| {2\pi eC\left( {\mathbf{X}} \right)} \right|$$

where e is Euler’s number. For the calculation of pairwise mutual information, configurational entropy is calculated for each residue of interest and each pair that can be formed between these residues. The mutual information for a pair of residues *A* and *B* is then given by:

3 $${\textit{mutual information}}\left( {A,B} \right) = H({\mathbf{X}}_{A} ) + H({\mathbf{X}}_{B} ){-}H({\mathbf{X}}_{A,B} )$$

In this study, the set of residues of interest was taken as the upper lid of the binding pocket, the loop immediately above this, the TRP-like domain, the helices of the voltage-sensor-like-domain immediately connected to these, and the transmembrane domain coiled coil actually containing V961 (Fig. 1A) These chain segments incorporate residues L663-Q676 (binding pocket lid (H4 in Suo et al.’s nomenclature^40^, see Fig. 1 C)), V961-V1005 (loop above pocket lid (H7), TRP-like domain and transmembrane domain helix (part of S6)), Y706-L730 (VSLD helix threaded through loop above pocket lid (S1)), and Y842-V865 (VSLD helix (S4)) and connected transmembrane (TMD) helix immediately adjoining V961 (S5)). All non-hydrogen atoms were used. A more complete residue breakdown is provided in SI Text S5. Coordinates were extracted from trajectory files by standard GROMACS tools; further analysis was done using locally written scripts. Structures were aligned by their TRP-like domains (Cα atoms) to provide a common reference framework. Molecular dynamics simulations based on the cryo EM structures 6PQQ and 6V9X were used to extract pocket-closed and pocket-open trajectories, respectively. Only these trajectories (i.e. simulations from conditions 1 and 3 in Table 1) are reported below. Analysis of 6PQP was performed, but this essentially gave almost identical results to 6PQQ and so is omitted for brevity.

## Results

### A stable open pore cannot be sustained with closed pockets

**Figure 3:**
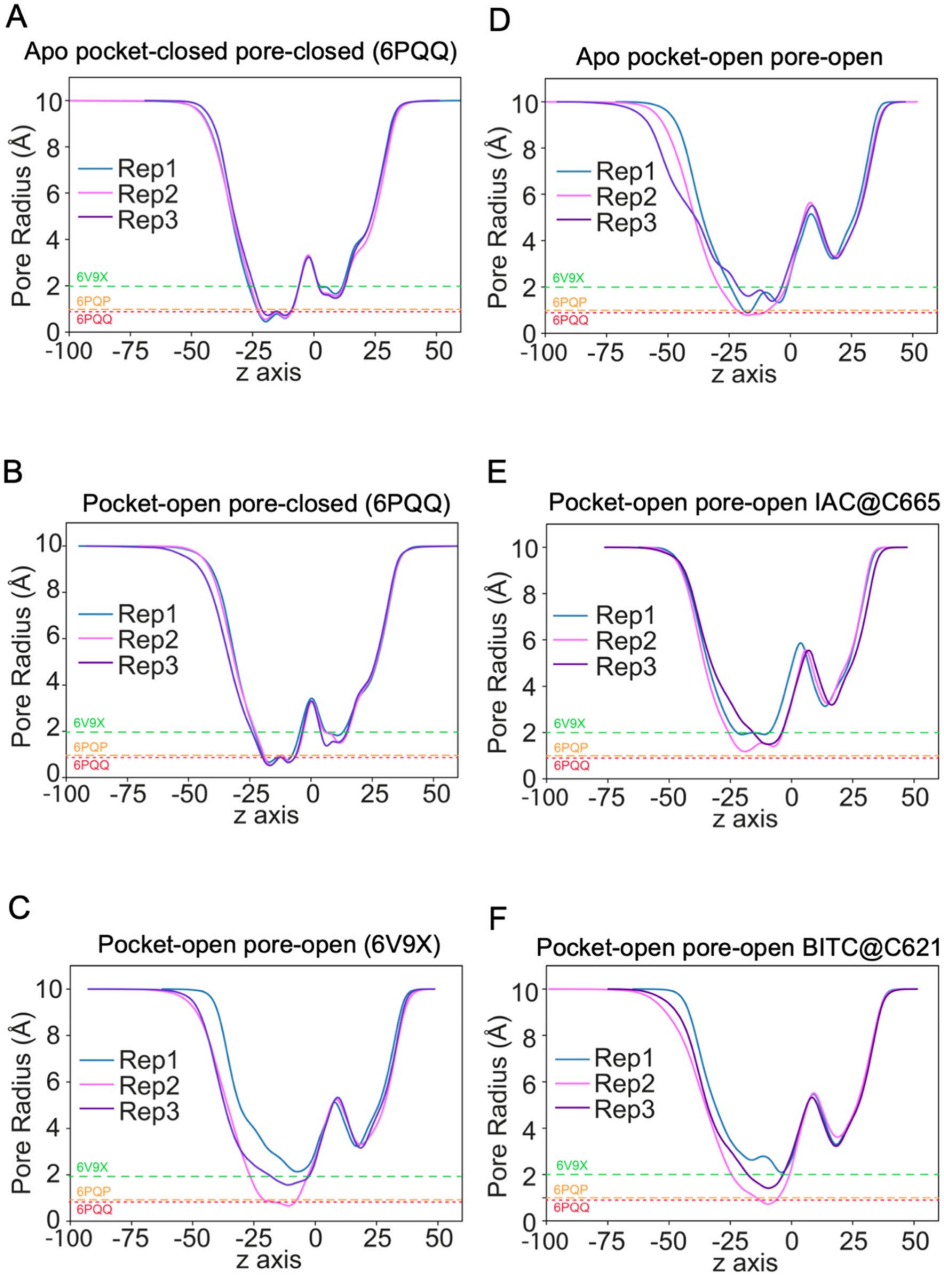
Average pore radius profiles for the 6 different systems simulated (see Table 1) as assessed by CHAP for (**A**) apo pocket-closed pore-closed (6PQQ), (**B**) pocket-open por-closed (6PQP), (**C**) pocket-open pore open (6V9X) with IAC bound at C621, (**D**) apo pocket-open pore-open, (**E**) pocket-open pore-open with IAC at C665 and (**F**) pocket-open pore-open with BITC at C621. Three repeat runs are indicated by blue, pink and purple lines. The minimum radii of the three crystal structures are also indicated for reference. Individual profiles with error bars (1 std dev) are shown in SI Figs. S2 and S3 and show no dramatic deviations.

In this work, we set out to better understand the relationship between the state of the pocket where electrophiles bind to (open or closed) and the conformation of the central channel pore. Thus, we examined the minimum pore-width (as assessed by CHAP^58^) as a function of time for all six initial conditions described in Table 1. The simulations reveal that only when a ligand is present in the pocket is the pore more likely to be stabilised in an open state (Fig. 3 and SI Figs. S2, S3). The central pore region in pocket-open pore closed (6PQP) and pocket-closed pore-closed (6PQQ) remains closed throughout the simulation (Fig. 3 and SI Figs. S2 and S3). The same result is seen in all repetitions of these simulations. The simulations for pocket-open pore-open (6V9X) with the native ligand (IAC at C621) show that in 2 of the 3 simulations the pore remains open, with only one of the repeats tending to closure (Fig. 3C and SI Figs. S2G–I). In the apo pocket-open pore-open structure, the pore is seen to close in all simulations (Fig. 3D and SI Fig. S3A–C).

Interestingly the pore profiles for when IAC is bound to C665 show the pore closes fully or partially in two repeats (Fig. 3E and SI Fig. S3D–F) leading to very similar profiles as shown for the pocket-open pore-open (6V9X) with no ligand simulations. However, when BITC is bound to C621 (pocket-open pore-open with BITC at C621) the pore-radius only shows full closure in one of the repeats (Fig. 3F and SI Fig. S3G–I) and indeed overall the profiles look similar to the pocket-open pore-open (6V9X) with IAC bound to C621 (Fig. 3C).

The implication of these observations is that (i) open pockets are required to sustain an open channel pore, but (ii) open pockets will not directly result in a closed channel pore shifting to an open state (at least not readily on this timescale). It is therefore particularly important to examine exactly how the presence of a ligand influences the open or closed state of a pocket.

### Pockets are dynamic but ligands supress pocket closure

**Figure 4:**
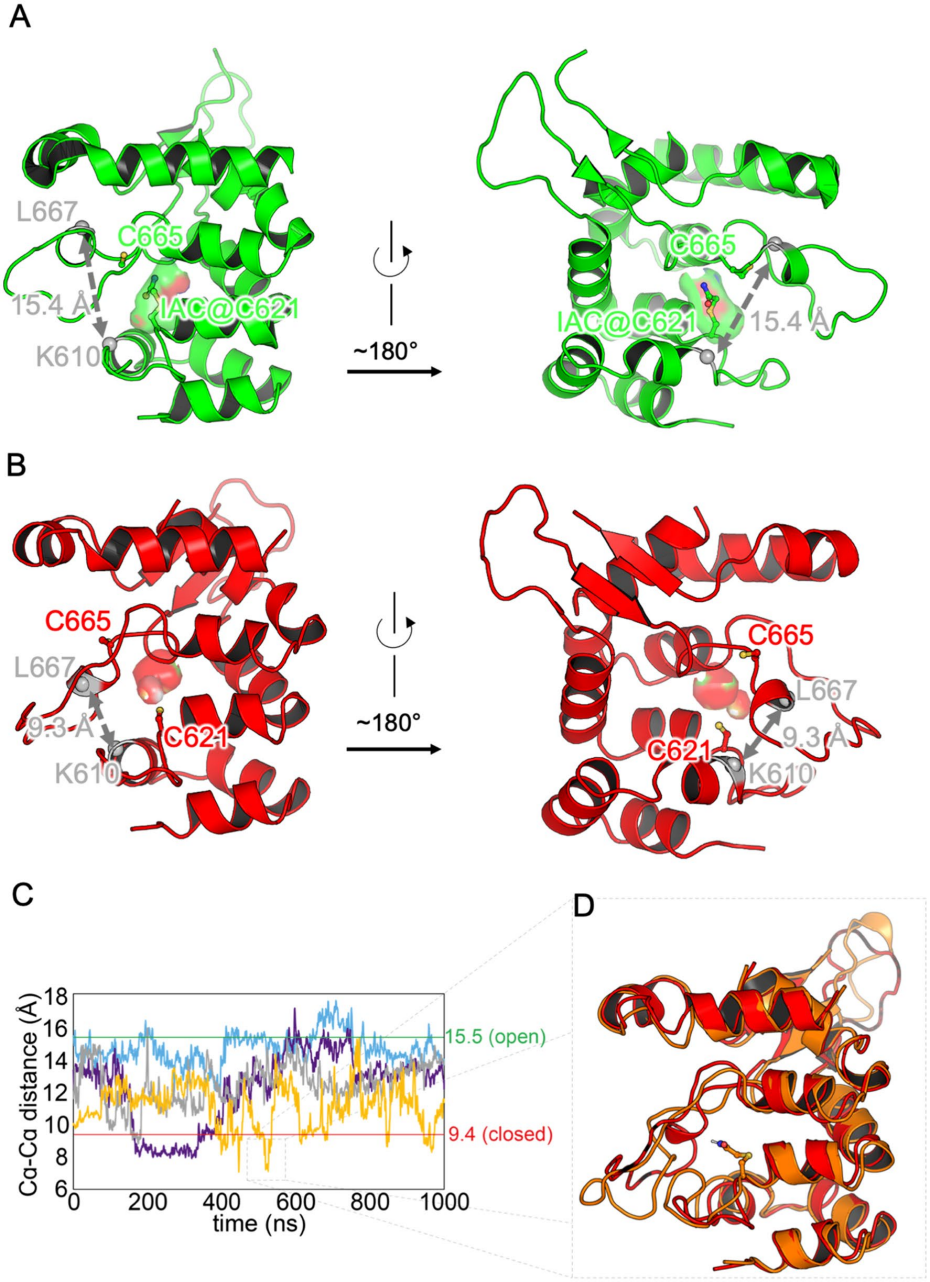
(**A**) The cytosolic domain binding pocket in the open (6V9X, green) and (**B**) closed (6PQQ, red) cryo EM structures. C_α_ atoms are shown for critical residues C621 (silver ball) and C665 (ribbon-coloured ball). IAC bound in 6V9X is shown as a ball-and-stick model, and residues Lys 610 and Leu 667 are shown as stick models. (**C**) Four example time series for pocket width (K610–L667 C_α_ − C_α_ distance) for pockets in Apo pocket-open pore-open simulation (condition 4 in Table 1) (purple and blue lines) and for pocket-open pore-open (6V9X) (condition 3 in Table 1) (grey and yellow lines). Values for cryo EM structures are shown as flat lines in green (6V9X, pore-open) and red (6PQQ, pore-closed). Moving averages over 2 ns are shown for clarity. (**D**) Binding pocket structure overlaid for cryo EM 6PQQ (red) and a snapshot from pocket-open pore-open with native ligand, IAC at C621 (orange). The pocket is closed in both cases.

Visual inspection of the simulations revealed that the lids of the pocket were quite flexible (consistent with the experimental difficulty in resolving this structure, resulting in its absence from cryo EM structures 6PQQ and 6V9Y). All simulations, with or without ligands, were observed to have pockets moving from open to closed states and back again. This was quantified via the distance between the C_α_ atoms of residues K610 and L667, on the lower and upper lids of the pocket, respectively. The pocket in open and in closed states, with these residues marked, is illustrated in Fig. 4A,B. This distance is 15.5 Å in pocket-open structures (6PQP, 6V9X) and 9.4 Å in pocket-closed structures (6PQQ). The time series for this distance is illustrated for four binding pocket simulations; 2 repeats from pocket-open pore open (6V9X) and 2 repeats from apo pocket-open pore open in Fig. 4C. Full pocket time series are shown in the SI Figs. S3 and S4.

As can be seen, in one of the four repetitions, the pocket remains open (blue), whereas the other three exhibit dynamic behavior and move from open to closed, and back again. These time series are typical (see SI Figs. S4, S5) of those observed in these simulations, both without ligands, and with ligands bonded in place (Fig. 4C). The closed-pocket conformations observed closely resemble the 6PQQ structure (Fig. 4C,D). The question arises of what proportion of time the pockets are closed in each simulation, and how far this influences the open or closed state of the channel pore. Using a cut-off of 11 Å on the C_α_ distance between K610 and L667, the proportion of time spent in closed states was calculated for each ligand-bonding scenario, over all three repeats and for all four monomers in each channel, giving a total of 12 μs simulation time for each ligand bonding scenario. The proportions and number of pore-open repetitions are reported in Table 2.

**Table 2:** Pocket-closed population based on K610–L667 C_α_ − C_α_ distance < 11 Å for ligand-bonding scenarios, and corresponding numbers of repetitions with open ion channel.

Structure and ligand	Ligand	Population closed (%)	Number of repetitions with open ion channel
6PQQ	None	88 ± 4	0
6PQP	BITC@C621	7 ± 5	0
6V9X	None	41 ± 9	1 Partially
6V9X	IAC@C665	22 ± 9	1 Fully, 1 partially
6V9X	IAC@C621	21 ± 7	2 Fully
6V9X	BITC@C621	6 ± 3	2

Thus, configurations with no bonded ligand (6PQQ, 6V9X with ligand deleted) have much higher proportions of closed pockets than other scenarios. As expected, the proportion is higher when the pockets are initialised in the closed state (6PQQ) than when they are initialised in the open state (6V9X with ligand deleted) – it is not clear whether, and over what time scales, these two proportions might converge and extensive further simulations would presumably be needed to address that directly. In both cases, the channel pore remains closed or partially closed for the vast majority of the simulation time. Conversely, the configurations with the largest bonded ligand (6PQP and 6V9X with BITC at C621) have very low—though non-zero—proportions of closed states (Table 2). The behaviour of the pore in both of these configurations is closely related to its starting state, staying open or partially open in 2 out of three repetitions for pocket-open pore-open with BITC at C621, and closed in all repetitions for pocket-open pore-closed (6PQP). Finally, the scenarios with a small bonded ligand—6V9X with IAC at C621, or at C665 (conditions 3 or 5, Table 1) show intermediate levels of pocket closure (which are the same as each other within error), and have respectively one and two repetitions out of three in which the channel pore closes fully or partially. However, it does not appear that channel pore closure results in a lower level of pocket closure: the single repetition with pore closure has 25% pocket closure when IAC is bonded to C621 and the fully closed repetition has 13% pocket closure (lower than the average for this scenario) when IAC is bonded at C665.

Taken together, these observations suggest that some degree of pocket opening occurs, even in the absence of ligands and starting from a closed pocket (*e.g.* in structure 6PQQ), but that the presence of a ligand strongly encourages pocket-open states, with larger ligands giving rise to higher proportions of pocket-open states (though not completely supressing pocket-closed states). An open channel pore is then stabilised by a high proportion of open pocket states, but this operates at a stochastic rather than a direct mechanical level, *i.e.* opening of one or more pockets does not directly push the pore open, but instead gives an existing open pore state a higher chance of stabilising in a given set of conditions. The degree of stabilisation is related to the proportion of pocket-open states. This is also consistent with the observation that open pockets do not lead to opening of an initially closed channel pore, as observed in 6PQP.

### Iodoacetamide bonded at Cysteine 665 facilitates pocket opening

**Figure 5:**
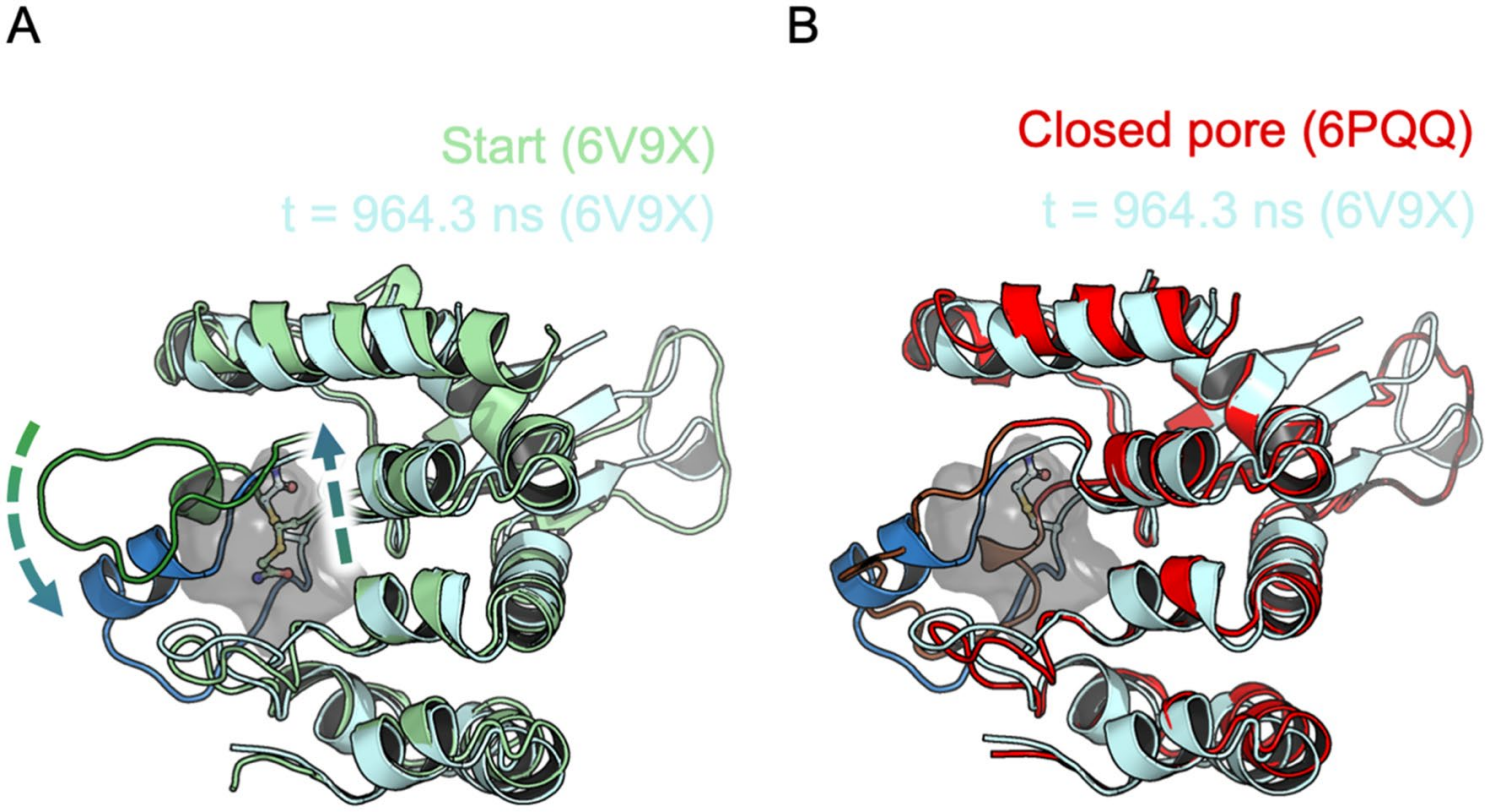
(**A**) Binding pocket structure overlaid for pocket-open pore-open with IAC at C665 (with ligand in ball-and-stick) at start (green) and at t = 964.3 ns (1st run, 2nd pocket by atom index) (pale blue) displaying rotation of IAC out of the pocket. The pocket also closes up as indicated by the change in conformation of residues 665–681 (show as dark green for 6V9X and dark blue for the snapshot). The pocket is indicated by the grey surface which encompasses all residues within 3.5 Å of the IAC nitrogen atom. (**B**) Comparison of the IAC out-of-the-pocket snapshot (pale blue with residues 665–681 in dark blue) with the cryo EM 6PQQ (red with residues 665–681 in brown)) highlighting similar backbone conformations and also showing clear intrusion of backbones into the pocket (grey surface).

Examination of trajectories reveals a crucial feature of IAC’s pose when bonded at C665. The ligand demonstrates the ability to rotate out of the pocket altogether in some instances of pocket closure (Fig. 5). The out-of-pocket pose for IAC bound at C665 was found to have occupancy of 3% over all 12 µs of pocket-simulation.

This is particularly significant, since C665 is solvent exposed when the pocket is closed, whereas C621 is not. This suggests a mechanism whereby a ligand can bond to C665 when the pocket is closed, then rotate into the pocket during a transitory opening event. The low occupancy of the out-of-pocket pose suggests that rotation into the pocket will be more stable for the ligand, and will also stabilise the open pocket. This will then expose C621 to the bulk solvent, facilitating bonding to that position as well.

### Mutual information pathway analysis suggests how the pocket is coupled to the pore

**Figure 6:**
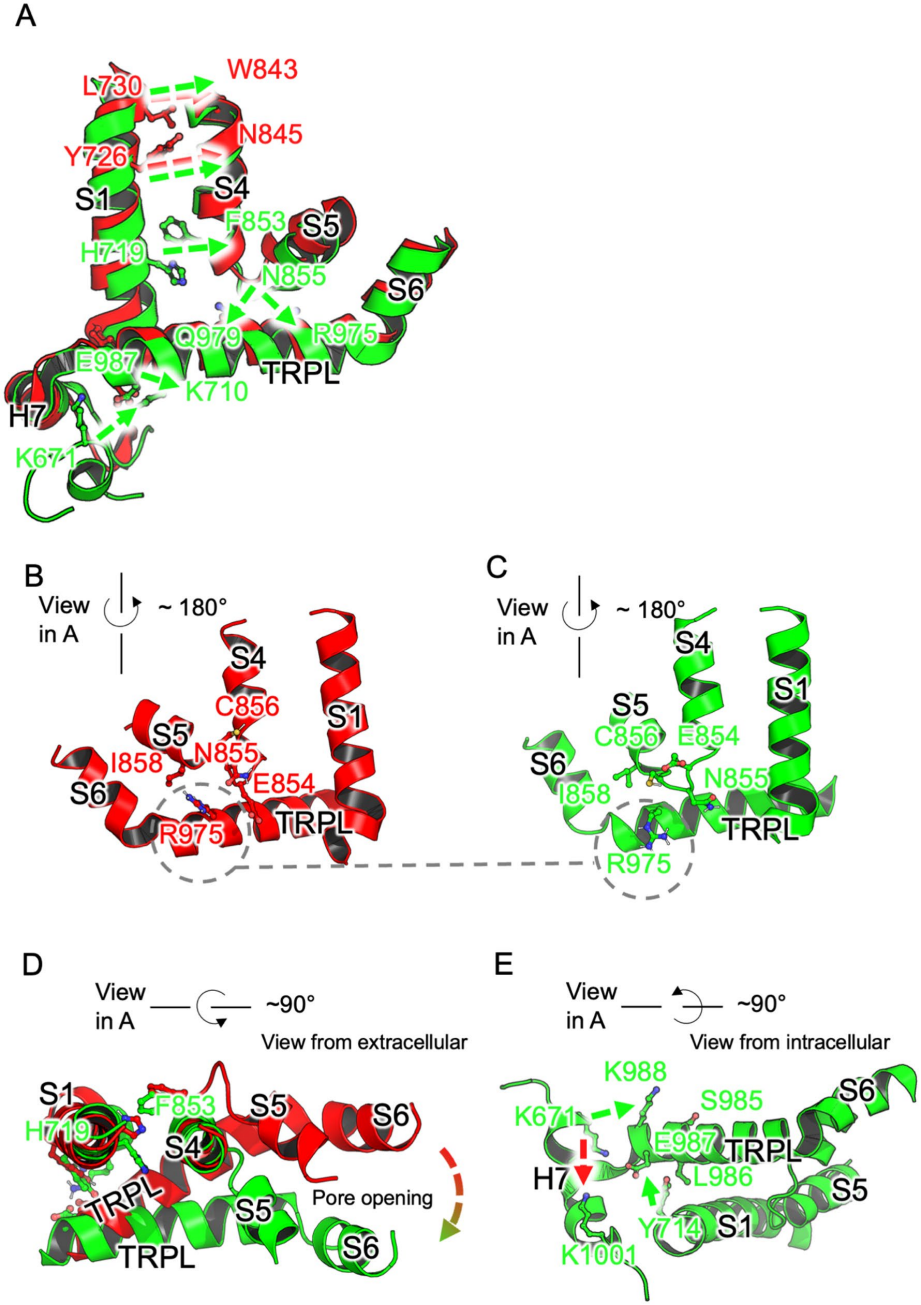
(**A**) Overlaid structures for pocket open pore-open (6V9X, green,) and pocket-closed pore-closed (6PQQ, red). The section of a single monomer unit between the pore (at V961) and the binding pocket lid (at K671, only present for pocket-open structure 6V9X) is shown. Strong pairwise mutual information exchanges are shown with green arrows for 6V9X and red arrows for 6PQQ. Side chains are shown only for 6V9X, except for Y726, N845, and V961 where 6PQQ side chains are shown for comparison. The protein segment is shown viewed across the plane of the monomer unit. (**B**) and (**C**) comparison of pocket- and pore-closed with pocket and pore-open structures highlight behaviour of R975 (grey dashed circles). (**D**) Close-up sections of 6V9X (green) and 6PQQ (red) showing S1, S4, S5, TRPL and S6, aligned on S4 emphasising the positional changes going from closed (red) to open (green). (**E**) Key residues identified by mutual information (only the 6V9X structure shown for clarity). K671 exhibits strong mutual information with K988 in the pocket- and pore-open simulations (green arrow), but stronger with K1001 in the pocket-closed pore-closed structure (red arrow).

Mutual information allows one to quantitatively assess the degree to which motion of one residue is correlated with motion in another. Chains of correlated pairs thus are indicative of coupling pathways within the protein. To elucidate a precise pathway of coupling between the dynamics of the pocket and pore-opening, we utilized the approach of LeVine et al. (see “Methods”) and focussed on key segments of the protein (a full description and list of residues used is provided in SI S6 Text). The maximum mutual information score between each residue and any other residue, and between each pair of chains as a whole was determined. The most prominent residue pairs from this analysis are summarised in Table 3. Figure 6A shows the pathways of strongest pairwise mutual information (judged by the maximum mutual information scores for each residue to any other residue) between the pocket lid and the channel pore in simulations initiated from cryo EM structures with pocket-open pore-open (6V9X) and pocket-closed pore-closed (6PQQ) (i.e. only the 3 repeat simulations from conditions 1 and 3 in Table 1 are discussed below). To compare sub-structures across this whole region, the structures are aligned on the TRP-like domain.

**Table 3:** Prominent residues identified via mutual information analysis.

Residue pair	Mutual information (6V9X)	Mutual information (6PQQ)
Arg975-Glu854	6.18	6.79
Arg975-Asn855	6.36	6.17
Arg975-Cys856	6.35	5.05
Arg975-Gly857	6.79	4.73
Arg975-Ile858	6.34	5.96
Phe853-His791	7.64	6.36
Tyr842-Tyr726	8.40	9.03
Tyr842-Leu730	7.36	7.98
Tyr714-Ser985	6.91	5.91
Tyr714-Ler986	7.48	6.86
Tyr714-Glu987	8.48	7.88
Tyr714-Lys988	7.03	6.17
Lys671-Lys988	5.31	3.68
Lys671-Lys1001	4.38	4.32
Lys672-Lys988	4.97	3.55
Lys672-Lys1001	4.41	4.12

In the pocket-open pore-open (6V9X) structure, the TRP-like domain and pore-lining helix, S6 (see Fig. 1C) that incorporates Val 961, interact with the adjacent helix pair and loop in the transmembrane domain (S4, S5 and connecting residues) via strongly correlated motion between R975 and the series of residues N855, C856, G857, and I858 (Fig. 6A) (maximum mutual information over all simulations of 6.36, 6.35, 6.79, 6.34, against a mean residue-pair maximum of 5.30). Maxima were taken over all pockets in all corresponding runs for a given residue pair, then means were taken over all residue-pair maxima in each pair of chain segments.

In the pocket-closed pore-closed (6PQQ) structure, the only comparably strong interaction is between R975 and E854 (6.79, against 6.18 in 6V9X), though the interaction with N855 (Fig. 6B) is retained to some extent (6.17 against 6.36 in 6V9X). In the pocket-open pore-open, structure 6V9X, the R975 sidechain is forced to adopt a conformationally restricted, partly folded conformation in order to avoid clashes (Fig. 6C).

The S4 helix itself interacts with the outer voltage-sensor-like domain helix S1 via strongly correlated motion between the residue pairs F853 and H719, and Y842 with Y726 and L730 (Fig. 6A) (maximum mutual information of 7.64, 8.40, 7.36 against a mean of per-residue maximum 6.17). In 6PQQ, the F853-H719 interaction in the ‘lower’ (intracellular-direction) part of S4 and S1 is much reduced (6.63, rather than 7.64).

The information transfer above, also leads to an ‘upward’ (extracellular) movement of the bulk of S5 in the open structure that leaves space for a similar movement by S6, opening the pore. The movement of S5 though, is also facilitated by a considerable realignment of the S4-S5 connecting loop (Fig. 6B,C and D).

Mutual information pathways are then found outwards from S1. In 6V9X, particularly high information exchange is found between Y714 and the set of residues S985, L986, E987 and K988 (Fig. 6E) (6.91, 7.48, 8.48, 7.03 against a mean per-residue maximum of 5.75) on the loop above the binding pocket (H7), with E987 having the strongest correlations (8.48). In the closed structure 6PQQ, this series of interactions is notably weaker (5.91, 6.86, 7.88, 6.17), and unlike the open structure, higher mutual information for these residues is found with Lys710 in several individual simulation repetitions (31 out of 48 possible instances for these four residues over twelve monomer repetitions). This is presumably an attractive interaction in the case of E987 (Fig. 6E).

Finally, in the open structure, K671 on the open pocket lid (Fig. 6E) is shown to engage in reasonably strong correlated motion with Lys988 (maximum mutual information of 5.31 against a mean per-residue maximum of 4.42), whereas when the pocket is closed, the strongest correlation between this residue and the upper loop H7 is with K1001 (mutual information of 4.32), at the opposite extreme of this segment. A similar though smaller shift is found for the adjacent K672 (4.97 with K988 in 6V9X, 4.12 with K1001 in 6PQQ).

**Figure 7:**
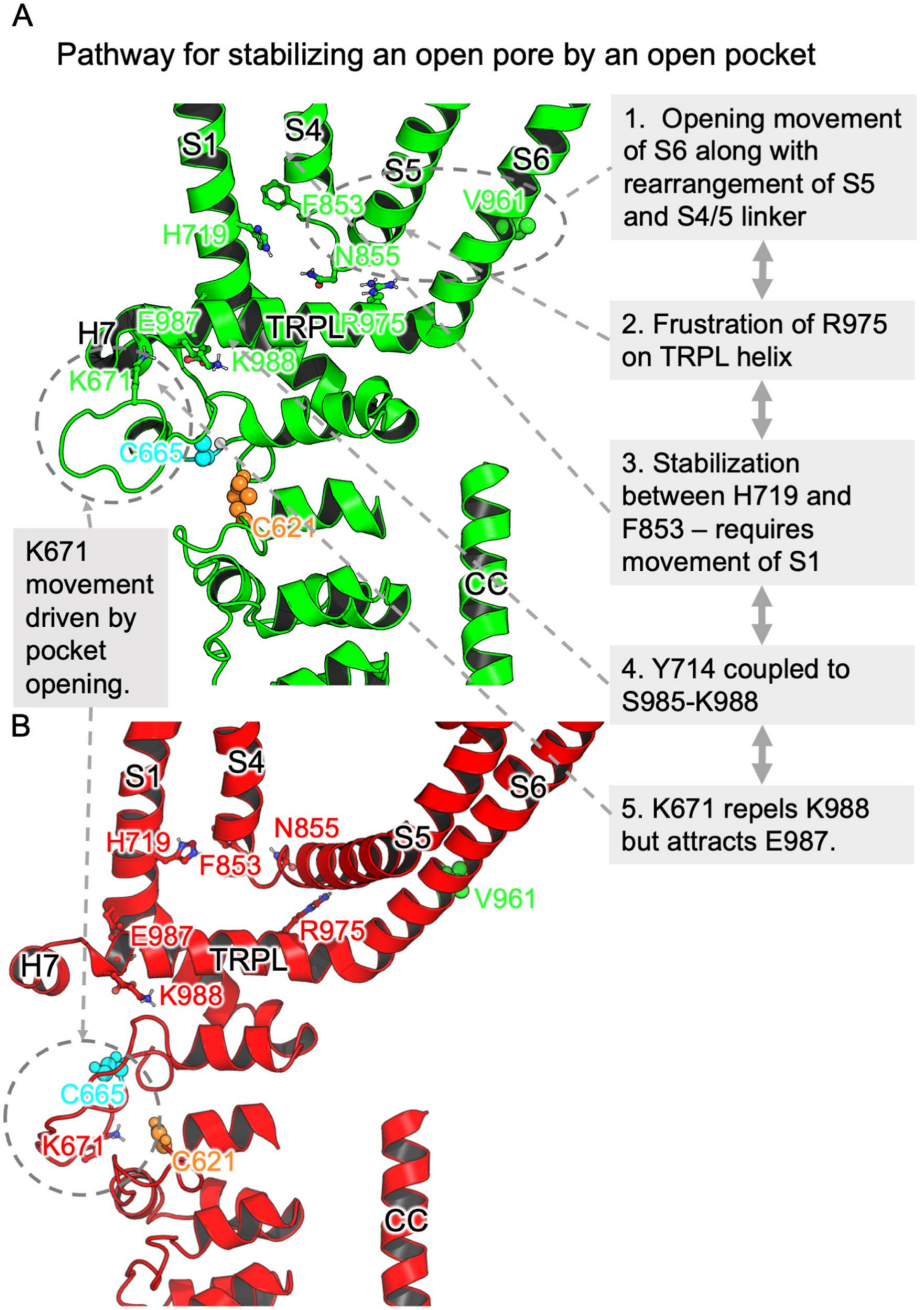
(**A**) Summary of the key interactions that help an open-pocket stabilize an open pore conformation as mapped onto the open state structure, 6V9X. (**B**) The closed state is provided for comparison in particular to show the key movement of the upper-lid of the pocket that allows interaction with the base of S1.

A complete mechanism for open-pore stabilisation by the open pocket can hence be reconstructed (Fig. 7). The upward movement of S6 that removes V961 from the channel pore requires space, which may arise from similar upward movement of S5 and commensurate rearrangement on the S4-S5 loop (Fig. 6D). However, this creates (or enhances) correlations between the N855-I858 segment on the S4-S5 loop and R975 on the TRPL-S6 chain. The conformational restriction (entropic reduction) and folded conformation imposed on R975 destabilises the open pore structure. This assumption is supported by the observation that a N855S mutation results in gain-of-function^60^. It then appears that stability is restored by the interaction between F853 and H719, facilitated by the rotation of F853 with the realignment of the S4–S5 linker. This interaction is between F853 and specifically the methylene group and side of the histidine ring with carbon atoms only, and hence is essentially hydrophobic. It also requires movement of the upper portion of the S1 helix, which is accompanied by the movement of Y714 at its lower hinge and an accompanying shift in the highly coupled segment, S985-K988 (with E987 particularly prominent). When the binding pocket is open, this movement is encouraged by repulsion between K671 and K988; when the pocket is closed, no such interaction is possible. Hence, the binding pocket lid is found to provide stabilisation at the terminus of a sequence of structural shifts that ultimately stretch from the space provided for channel pore opening at the center of the channel to the exterior face of the protein (Fig. 7).

The prominent role of K671 is in agreement with the mechanism proposed by Zhao et al.^38^ There is also some consistency with the observed phenomenon^36^ by which IAC substitution at K710 provides limited activity even when C621 and C665 have been mutated to nonreactive residues. Interaction with K710 seems to be more significant (highest mutual information between two chain segments in more cases) for E987 in the closed structure than in the open structure; this is suggestive of an attractive interaction stabilising the closed structure relative to the open structure. Bonding of IAC to K710 may lower this interaction, hence shifting the balance towards the open structure.

## Discussion

The results reported here represent significant progress towards answering question (i) as posed in the Introduction: how does the open or closed state of the pocket influence the opening and closing of the channel pore? The tendency to closure of an initially open channel pore in the presence of high proportions of closed pocket states, suggests that open pockets stabilise an open pore. Mutual information pathway analysis confirms this – an open pore is accompanied by shifts in the surrounding structure, moving outwards from the pore at the core of the channel structure to the H7-S1 complex at the exterior of the channel. The open binding pocket lid then supplies the K671 residue to help lock in these new positions of residues in this region. This differs from the proposal by Zhao et al.^38^ that electrostatic interactions between the pore-adjacent termini of TRP domains in neighbouring monomers serve to stabilise the open-pore state; no significant mutual information was measured between these termini in this work, suggesting that this factor may be less influential. The only part of the question left unresolved is whether the pocket plays any role in the initial opening of the pore, and whether any other factors are involved in that initial opening. It can be speculated that the pore undergoes occasional transitory dilations whether or not the pockets are open; but that in the presence of open pockets, these will be sufficiently stabilised to shift from transitory to stable. The opening of the pore would then be entirely dictated by the open or closed state of the pockets, without the need for any additional factor. If this is the case, it may occur on timescales longer than were accessed in this work.

This work has established that bonded ligands, whether at C621 or C665, serve to suppress the population of pockets in a closed state, which in turn help to stabilise the open pore state. This observation suggests that in the purposeful design of electrophile agonists for TRPA1, not just potential interactions with the open pocket, but also potential interactions with the closed pocket (as a sort of ‘anti-target’) must also be considered, whether the molecule can be accommodated in a minimally flexed closed pocket, or could project into the adjacent solution. Another aspect of the relationship between ligands and opening of pockets is suggested by question (ii) as posed in the Introduction: the role of bonding at C665. Zhao et al. have suggested^38^ a ‘two-stage’ mechanism for TRPA1 activation by IAC in which bonding at both C621 and C665 is necessary to open the pocket and hence the pore, due to the small size of the ligand. It is further suggested that such simultaneous bonding would not be necessary (or possible) for larger ligands. This work has shown (Table 2) that bonding of IAC at either C621 or C665 gives a similar degree of suppression of pocket-closed states, which is smaller than that provided by the large ligand BITC; and this appears to result in a lower level of open-pore stabilisation for single-IAC bonding than is given by BITC. Bonding at both sites would therefore be expected to give better stabilisation of an open pore, consistently with Zhao et al.’s hypothesis, and this may be necessary to give full activation of the channel on longer timescales, for small ligands such as IAC. The observation by both Bahia et al. and Zhao et al. that IAC bonding at C665 occurs at very low levels (3%), though, makes this explanation somewhat unsatisfactory: if simultaneous bonding is necessary, how is activation achieved with a maximum of 3% occupancy?

An alternative explanation is provided by the observation in this study that IAC bonded at C665 can rotate into and out of the pocket as it opens and closes. Additionally, it is simple to show that C665 is accessible by the bulk solvent when the pocket is closed whereas C621 is not. It can therefore be surmised that IAC initially associates with a closed pocket by bonding at C665. During a transitory pocket opening (as seen even in simulations of 6PQQ), the ligand then rotates in and stabilises the open pocket structure. With C621 now accessible, it is likely that either another electrophile will bond to it, or that the original electrophile will transfer, either directly or by returning briefly to solution. Hence, bonding at C665 would be vital to pocket opening and hence channel stabilisation, while only remaining as an occupied state in a small proportion of pockets. This hypothesis is not straightforwardly compatible with the observation of Bahia et al*.* that bonding to C621 still occurs even when C665 is mutated to leucine, which would suppress initial pocket opening in the scheme under discussion. We speculate that the relatively high concentrations and long timescales used in the study of Bahia et al*.* allow for bonding of small electrophiles even to C621 in the closed pocket, against considerable kinetic barriers, perhaps during the kinds of relatively uncommon opening events observed in the simulation of 6PQQ. It is not clear if the ideas above would apply to larger electrophiles, such as BITC.

We should also note that the discussion above and interpretation of our results, is based on finite 1 µs unbiased MD simulations. We cannot rule out the possibility that there are different, longer-time scale rearrangements that we have not observed here yet can account for the experimental observations. Much longer, or rather, a larger number of repeat simulations would likely be necessarily to definitively rule that out. Such an amount of data would also enable techniques such as Markov state modelling^61^ to be successfully constructed (there is not enough data in this study to successfully do this here) and this is something we are exploring in future work.

## Conclusions

It is well-established that electrophile activation of TRPA1 (and hence the channel’s chemosensation) is based around covalent binding to one of two cytosolic cysteine residues, C621 and C665. Recent structural data has revealed that these lie in a pocket-like structure. The structure of bonded electrophiles has been determined at C621; these are incorporated into pocket-open protein structures, whereas *apo* structures are pocket-closed. An open channel pore is only observed in the presence of open binding pockets, and hence a bound electrophile. However, the mechanism by which an open pocket leads to an open channel pore was not previously known, and the role of bonding to C665, experimentally established to occur with low occupancy, was not clear.

We have carried out 1 µs simulations of TRPA1 structures with pocket-closed pore-closed, pocket-open pore-closed, and pocket-open pore-open configurations, based on the cryo EM structures 6PQQ, 6PQP, and 6V9X, respectively. These simulations demonstrate that initially open pockets shift to a high occupancy of closed states in the absence of a ligand, and can reach a ~ 20% closure population even in the presence of a small ligand; that closure of an initially open channel pore will occur in the presence of highly-populated closed states, and has a chance of occurring in the presence of lower closed populations; and that open pockets will not result in opening of an initially closed pore on the timescales simulated here. The role of the open pockets therefore appears to be stabilisation of an already-opened pore.

Pairwise mutual information analysis of interactions between residues in the subdomains between the channel pore and the pocket reveals that opening of the pore results in a series of small structural shifts outwards from the core to the exterior of the channel, supported by a novel set of interactions that are not found in the closed channel. The external terminus of this chain of interactions is stabilised by interactions with the lid of the opened binding pocket.

The role of binding IAC at C665 has been clarified. IAC is capable of bonding to this residue even when the pocket is closed, and can then rotate into a more stable position within the pocket when it opens. The more reactive C621 residue then becomes solvent accessible, allowing for formation of the final, more stable complex. Binding to C665 has therefore been suggested as being essential to the initial opening of the pockets but not to them remaining open, explaining the low degree of activation found in C665 mutants despite the fact that C665 is found to have very low levels of bonding in the final products. It is not yet clear whether this mechanism is also relevant to larger electrophiles closer to the size of the pocket such as BITC or JT010. We look forward to experimental work to help clarify this issue.

## Supplementary Information

Supplementary Information.
