# Supplementary material for: Atomistic mechanisms of human TRPA1 activation by electrophile irritants through molecular dynamics simulation and mutual information analysis
Source: Sci Rep. 2022 Mar 23;12:4929. doi: 10.1038/s41598-022-08824-7 (PMC8943162; doi:10.1038/s41598-022-08824-7)
Supplement: Supplementary file 1 — Supplementary Information. [file 41598_2022_8824_MOESM1_ESM.pdf]

SUPPLEMENTARY INFORMATION

Atomistic mechanisms of human TRPA1 activation by electrophile irritants through molecular dynamics simulation and mutual information analysis

Matthew Habgood[1,2*], David Seiferth, Afroditi-Maria Zaki[1], Irfan Alibay[1], and Philip C. Biggin[1*]

[1]Department of Biochemistry, University of Oxford, South Parks Road, Oxford. OX1 3QU. UK.

[2]AWE Aldermaston, Reading, Berkshire, RG7 4PR. UK.

UK Ministry of Defence © Crown Owned Copyright 2021 / AWE

Fig S1. Individual RMSD time series of the backbone atoms for each repetition. RMSDs are shown for simulations based on experimental structures pocket-closed pore-closed (6PQQ) (**A-C**), pocket-open pore-closed (6PQP) (**D-F**) and pocket-open pore-open (6V9X) (**G-I**).

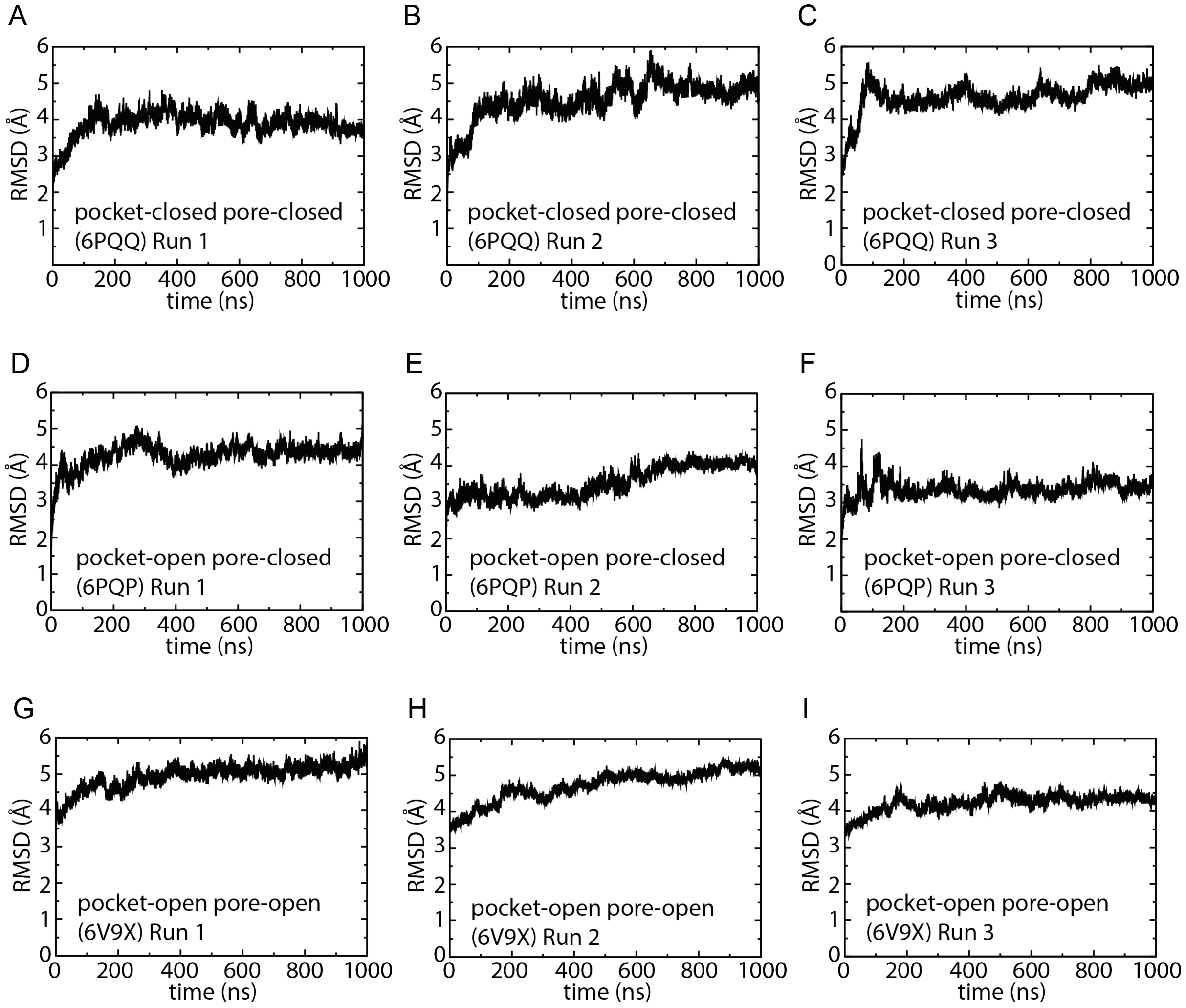

Fig S2. Pore radius profiles. Pore radius (computed for the transmembrane region only) as a function of the *z* coordinate within the simulation box are shown for simulations based on experimental structures for pocket-closed pore-closed (6PQQ) (**A-C**), pocket-open pore-closed (6PQP) (**D-F**) and pocket-open pore-open (6V9X) (**G-I**). V961, which is considered as the main gating residue, is located at z = -24 Å.

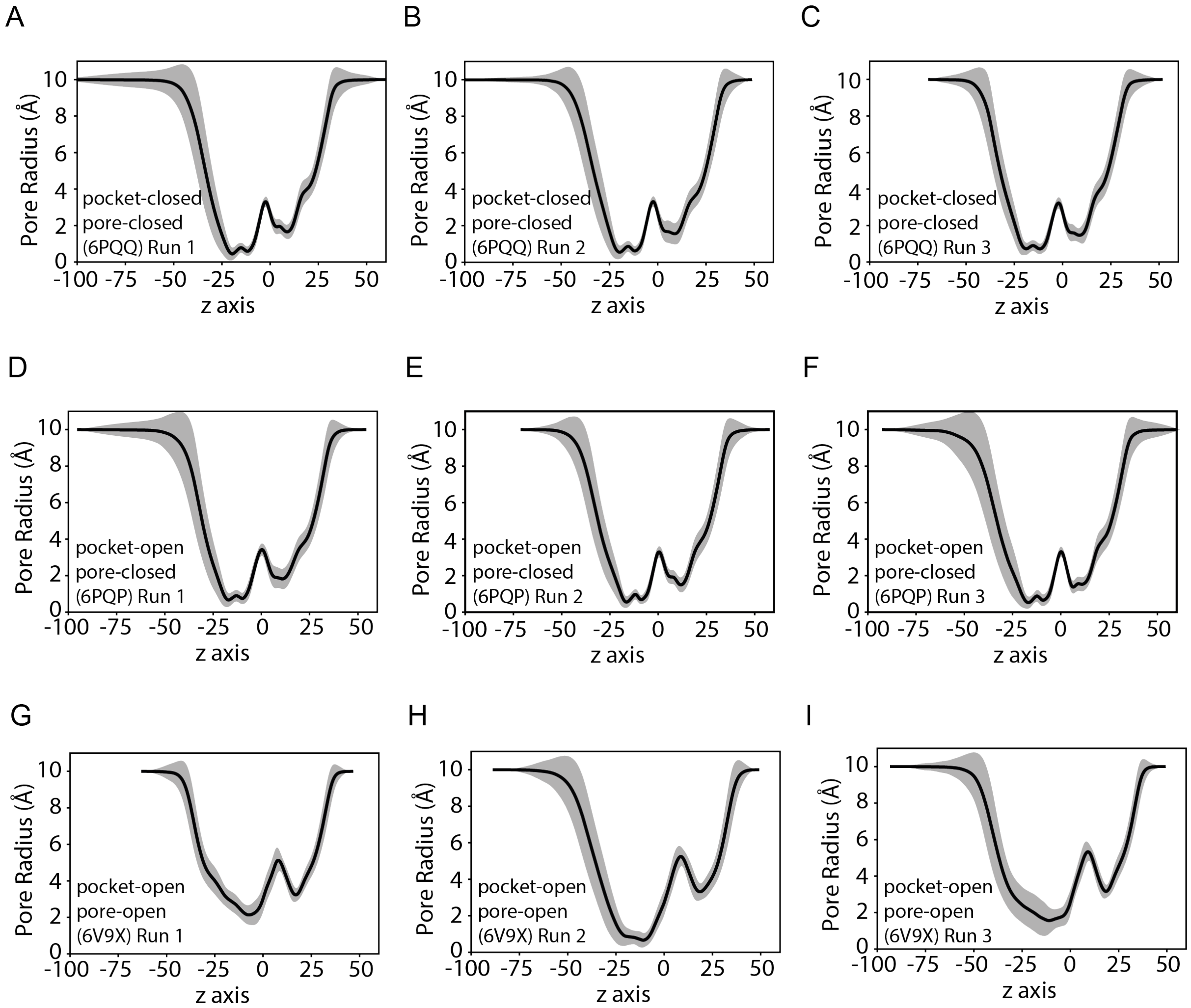

Fig S3. Pore radius profiles for 6V9X in various alternative conditions. Pore radius (computed for the transmembrane region only) as a function of the *z* coordinate within the simulation box are shown for simulations for Apo pocket-open pore open (**A-C**), pocket-open pore open with IAC at C665 (**D-F**) and pocket-open pore open with benzyl isothiocyante (BITC) at C621 (**G-I**). V961, which is considered as the main gating residue, is located at *z* = -24 Å.

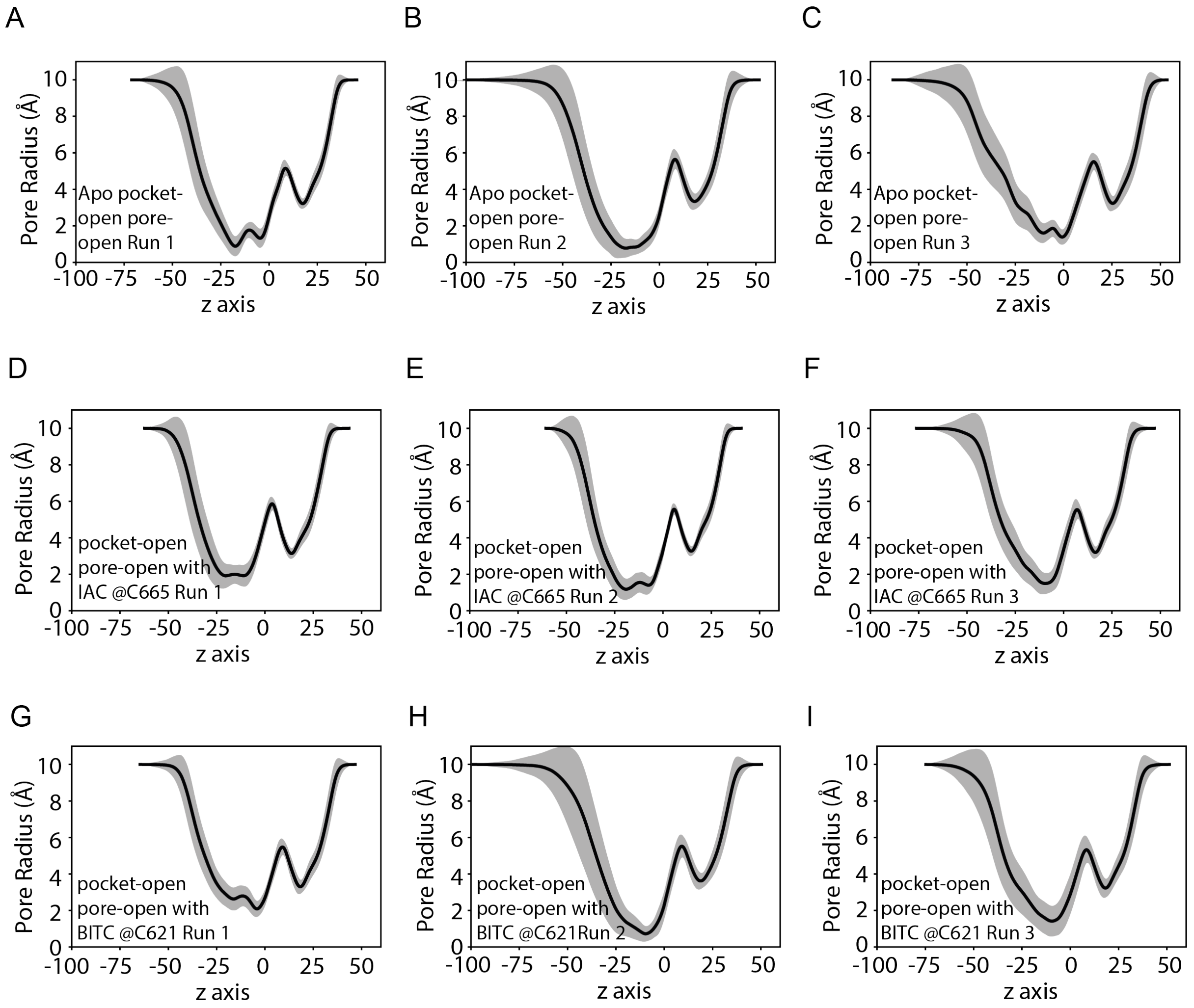

Fig S4. Pocket width time series. The width of the pocket as indicated by the C_α-C_α distance between residues Lys610 (lower lip) and Leu667 (upper lip) for pocket-closed pore-closed (6PQQ) (**A-C)**, pocket-open pore-closed (6PQP) (**D-F**) and pocket-open pore-open (6V9X) (**G-I**). Corresponding distances in 6PQQ and 6V9X structures are 9.4 Å and 15.5 Å, respectively.

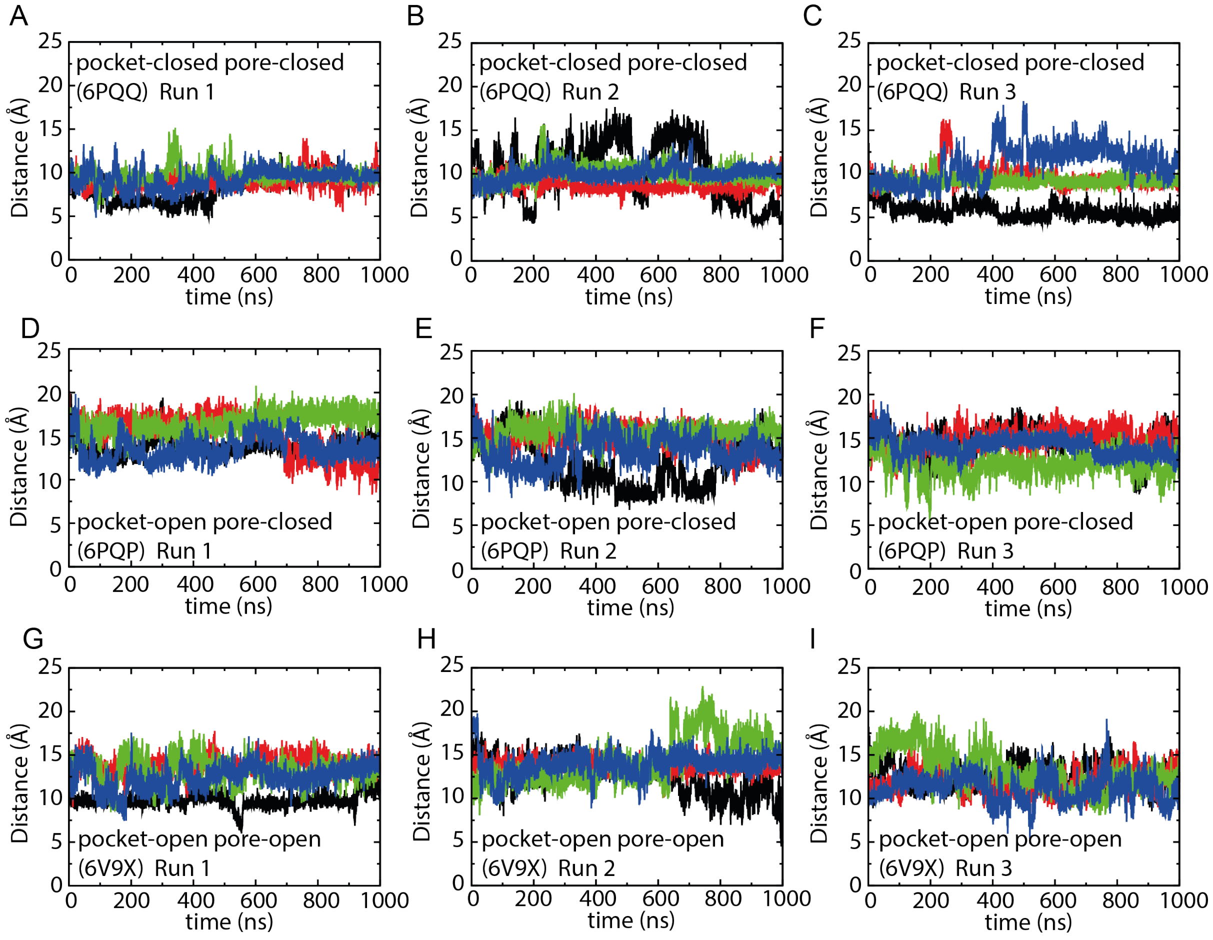

Fig S5. Pocket width time series for 6V9X in various alternative conditions. The width of the pocket as indicated by the Cα-Cα distance between residues Lys610 (lower lip) and Leu667 (upper lip) for Apo pocket-open pore open (**A-C**), pocket-open pore open with IAC at C665 (**D-F**) and pocket-open pore-open with benzyl isothiocyante (BITC) at C621 (**G-I**). Corresponding distances in 6PQQ and 6V9X structures are 9.4 Å and 15.5 Å, respectively.

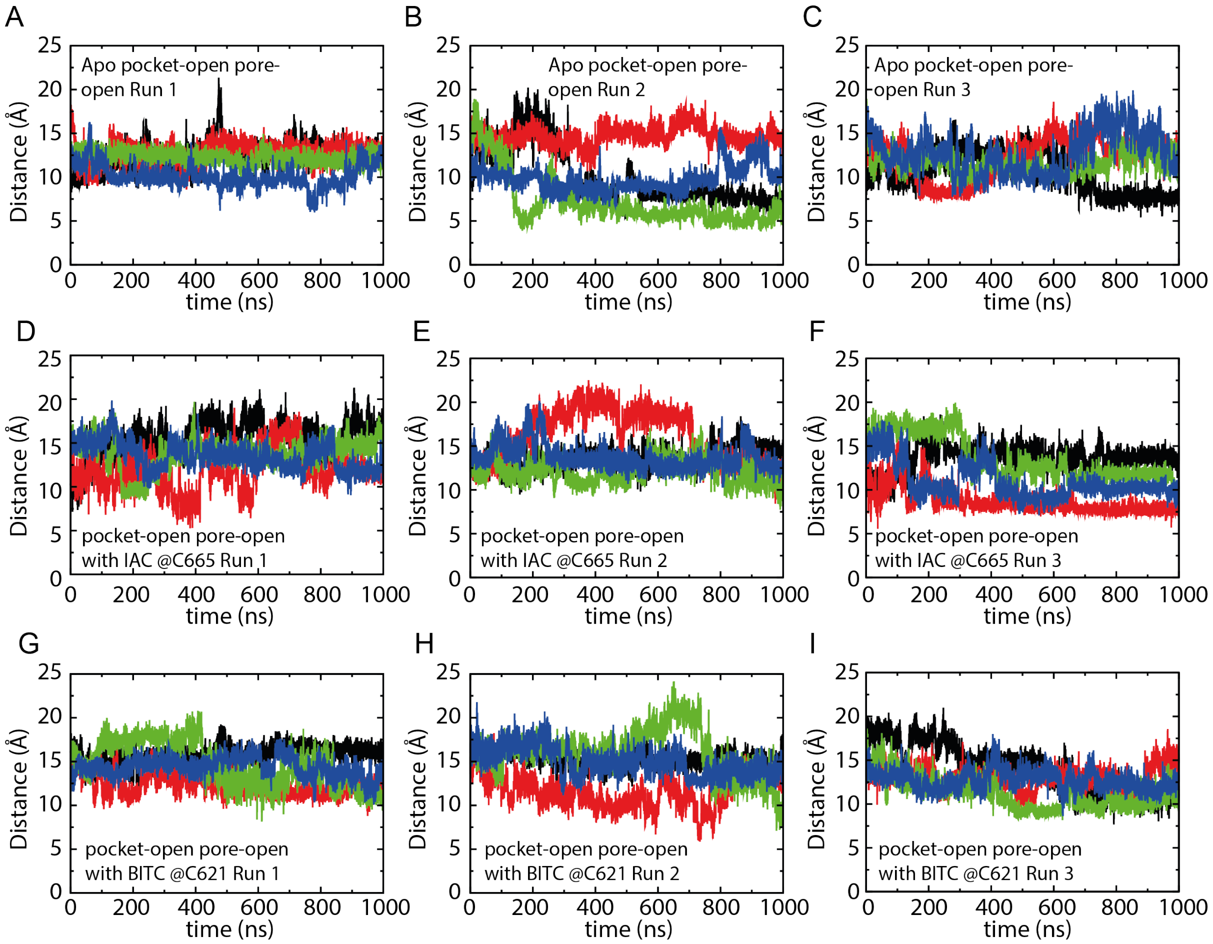

Text S6. Mutual information data

Pairwise mutual information was calculated between the residues of a set of chain segments in the region between the cysteine 621 ligand binding pocket, and the ion pore. As shown in the main paper, Figure 1A, these segments are: the ligand binding pocket lid (H4) (Gln664-Thr684); the loop above the pocket lid (H7), TRP-like domain and transmembrane domain helix (part of S6) (Val961-Lys1001) ; the VSLD helix threaded through loop above pocket lid (S1) (Tyr706-Val737); and the VSLD helix (S4) and connected TMD helix immediately adjoining Val961 (S5) (Gln831-Val861).

For analysis purposes, these were further split down into seven sub-segments:

Segment 1: the binding pocket lid (H4) [Gln664 – Thr 684]

Segment 2: the loop above the binding pocket lid (H7) [His983 – Lys1001]

Segment 3: the section of the outer upright in the VSLD adjacent to segment 2 (S1) [Tyr706 – Tyr714]

Segment 4: the upper part of the outer upright in the VSLD (S1) [His719 – Val737]

Segment 5: the inner upright in the VSLD (S4) [Gln831 – Phe 853]

Segment 6: the loop in the transmembrane domain, connected to S4, adjacent to the TRP domain and pore-bordering helix (S5) [Glu854 – Val861]

Segment 7: the TRP domain and pore-adjacent helix (S6) [Val961 - Gln979]
